# An Unusual Case of Inguinal Hernia With the Left Ovary and an Ectopic Left Pelvic Kidney in a 37-Year-Old Woman: A Unique Clinical Encounter

**DOI:** 10.7759/cureus.50251

**Published:** 2023-12-09

**Authors:** Kavyanjali Reddy, Pankaj Gharde, Harshal Tayade, Mihir Patil, Lucky Srivani Reddy

**Affiliations:** 1 Department of Surgery, Jawaharlal Nehru Medical College, Datta Meghe Institute of Higher Education and Research, Wardha, IND; 2 Department of Obstetrics and Gynaecology, Jawaharlal Nehru Medical College, Datta Meghe Institute of Higher Education and Research, Wardha, IND

**Keywords:** reproductive age, ectopic kidney, surgical intervention, ovarian herniation, inguinal hernia

## Abstract

Gynecological components, including ovaries, fallopian tubes, ligaments, and the uterus, are seldom found within hernial sacs. The occurrence of groin hernias containing elements of female genitalia is not well-documented. This case report presents a 37-year-old woman with a unique clinical scenario involving an inguinal hernia containing the left ovary and an associated ectopic left pelvic kidney. The patient's clinical history, characterized by left inguinal pain and swelling, is detailed, including relevant reproductive and medical background. The diagnostic journey encompasses ultrasound and contrast-enhanced computed tomography, revealing the left-sided inguinal hernia with the left ovary. The report emphasizes the challenges posed by the coexistence of inguinal hernia, ovarian involvement, and ectopic pelvic kidney. A multidisciplinary approach is highlighted, encompassing surgical and medical considerations. Surgical intervention involves left-sided inguinal hernioplasty, with a particular focus on fertility preservation through the careful repositioning of the ovary. Postoperative care and considerations for successful recovery are discussed. In conclusion, this case report sheds light on the intricacies of managing a complex clinical presentation, providing insights into diagnostic, surgical, and postoperative aspects. The rarity of such cases underscores the need for ongoing research and collaborative discussions within the medical community.

## Introduction

An abnormal protrusion of an intra-abdominal or extra-peritoneal organ, or a part of it covered by a sac, through a defect in the myofascial plane of the abdominal wall's oblique and transversalis muscles into the inguinal region is known as an inguinal hernia. This frequently occurring, non-threatening surgical condition accounts for around 75% of all hernias affecting the abdominal wall [1]. The likelihood of developing an inguinal hernia over one's lifetime fluctuates, with approximations ranging from 27% to 43% for men and from 3% to 6% for women [2]. During the 12th week of gestation, an outpouching of the parietal peritoneum becomes prominent and it is called the process vaginalis. If the process vaginalis fails to close, a canal of Nuck is formed. It extends through the inguinal canal to the labia major. Anomalies of the canal of Nuck lead to hernia and hydrocele. Hernia of the canal of Nuck leads to complications such as strangulation, ovarian torsion, and incarceration. Hence, timely imaging, diagnosis, and intervention are of utmost importance.

In females, inguinal hernias are infrequent, constituting only 5% of reported cases. Despite their rarity in women, it is essential to conduct investigations to exclude complications such as hernia obstruction and strangulation, which can result in the necrosis of the involved organ. Inguinal hernia surgery is one of the most frequently performed procedures on surgical wards. The severity of symptoms associated with inguinal hernias varies across a spectrum, ranging from asymptomatic cases to instances of severe abdominal pain attributable to strangulation [3].

There are several organs that can protrude through the inguinal canal, including the bladder, colon, small bowel, and less frequently, the appendix or female adnexa. The prevalence of groin hernias involving components of the female genitalia is not well documented. In a retrospective study by Gurer et al., only 2.9% (7 out of 1,950) of patients undergoing hernia repair between 1989 and 2004 were identified as having hernias containing the ovary and fallopian tube [4]. These events are most often seen in pediatric cases and are often associated with congenital abnormalities. Given the rarity of this clinical disease in women of reproductive age group, it is imperative to keep a high degree of clinical suspicion in order to protect fertility and spot any associated genital abnormalities.

Inguinal hernias are a common and usually benign condition in the general population. Repairing inguinal hernias is a significant part of frequently performed surgical procedures [5]. Sometimes, unique structures within the abdominal area may be discovered within a herniated sac. Gynecological elements like ovaries, fallopian tubes, ligaments, and the uterus have been identified as infrequent contents of an inguinal hernial sac. These events account for approximately 6-7% of inguinal hernias in this particular patient category, with a higher frequency occurring in female newborns and young girls. These results are frequently associated with aberrant genital tract development. While it is not uncommon in young girls to have an indirect hernia containing an ovary that is frequently caught and cannot be pushed back, it is less frequent among women who are of reproductive age.

This case report tells a unique story about a 37-year-old woman whose condition is not what doctors usually expect with inguinal hernias. It goes beyond the usual understanding of this problem because, in this case, the hernia contains the left ovary. Simultaneously, there is also an incidental finding of the left pelvic kidney that was not in its usual position. This unusual combination gives us a rare look into how different medical issues can happen simultaneously in one person.

In this instance, we present an uncommon occurrence of inguinal hernia containing the ovary in a female of reproductive age. The revelation of the left ovary as the hernia content, compounded by the unexpected discovery of an ectopic left pelvic kidney, emphasizes the uniqueness of this case. This case report adheres to the Surgical CAse REport (SCARE) 2023 criteria [6].

## Case presentation

**Figure 1:**
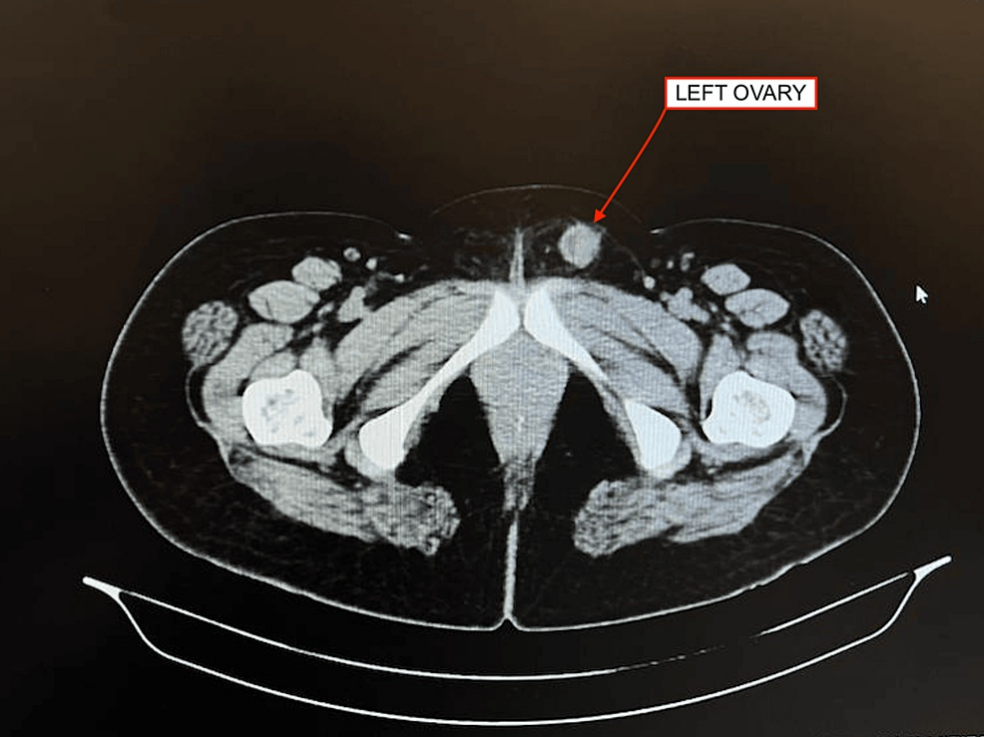
CECT of abdomen and pelvis confirms left-sided inguinal hernia with the left ovary and its mesovarium as content CECT: Contrast-enhanced computed tomography

A 37-year-old woman, belonging to a low socio-economic background, came to the surgery outpatient department with complaints of pain in the left inguinal region for six months. The pain was non-radiating in nature. She also complained of on-and-off swelling in the left inguinal region, which was mostly prominent before her menstruation and is associated with dragging sensations. The patient attained her menarche at 13 years of age. The patient has 3-4 days of regular menstruation, occurring at 28-30 days. The patient had two full-term, uncomplicated normal vaginal deliveries, one 14 years ago and the other 13 years ago, respectively. The past medical history was unremarkable, and the patient had no relevant family history. She had no bowel or bladder complaints. On general examination, the patient's general condition was fair, with no pallor, icterus, or clubbing. Her pulse rate was 72 beats per minute and her blood pressure was 120/80 mmHg. On a systemic examination, the respiratory and cardiovascular systems were found to be within normal limits. The patient was conscious and coherent. On per-abdominal examination, it was found to be soft, and non-tender, with no guarding, rigidity, or distention. Bowel sounds are appreciated in all four quadrants. On examination of the inguinal region, the bilateral inguinal region appeared normal, with no skin changes or visible swellings. The swelling was not appreciated even with coughing, straining, or standing positions, which made the clinical diagnosis of hernia very challenging. On palpation, there was no local rise in temperature or tenderness. A cough impulse was felt on palpation at the left deep inguinal ring. A vaginal examination revealed a normal, healthy cervix and vagina. Bilateral fornices were free with no cervical motion tenderness, and no per-vaginal white discharge or bleeding was noted. An ultrasound of the abdomen and pelvis was done, which was suggestive of the fact that the left kidney is not visualized in the left renal fossa. The left kidney is noted in the left iliac fossa, measuring 9.3x5 cm, with normal size, shape, and echotexture. It also showed a left inguinal hernia with a soft tissue mass with a few small cysts and a herniated ovary within the left inguinal region; the other solid organs and genital organs showed normal findings. Subsequently, contrast-enhanced computed tomography (CECT) of the abdomen and pelvis was done, which confirmed a left-sided inguinal hernia with the left ovary and its mesovarium as content (Figure 1).

**Figure 2:**
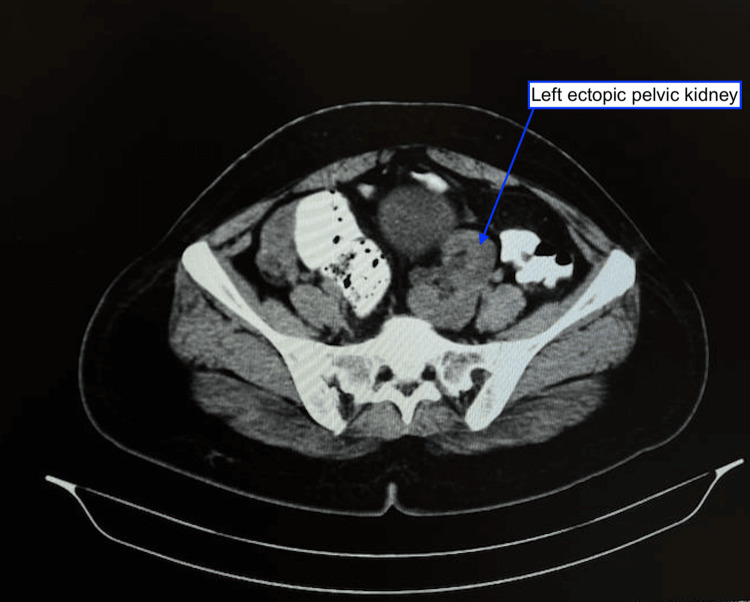
CECT of the abdomen and pelvis confirms the presence of a left ectopic kidney located in the left paramedian pelvic region, with the hilum facing anteromedially CECT: Contrast-enhanced computed tomography

The CECT of the abdomen and pelvis also revealed an additional observation of the left ectopic kidney. It was noted in a left paramedian pelvic region, measuring approximately 9.5x3.9 cm, with the hilum facing anteromedially (Figure 2). The left kidney is supported by a branch of the artery arising at the level of the upper-end plate of the L4 vertebra from the left common iliac artery. A rounded mixed echogenic structure measuring 2.2x5.5x4.3 (27.8 cc) is seen in the left inguinal canal, with few cystic areas within the likely follicles. The dominant follicle measures 1.7x2.9 cm. The hemorrhagic follicle measures 1.8x2.9 cm.

All routine blood and urine examinations were conducted, and the results of all tests were within normal limits (Table 1).

**Table 1:** Investigation profile of the patient

Investigations	Patient	Reference values
Hemoglobin	14 g/dl	12-15 g/dl
Total leukocyte count	11500/dl	4000-11000/dl
Platelet count	371,000/dl	150,000-400,000/dl
Serum creatinine	0.6 mg/dl	0.5-1.2 mg/dl
Albumin	4.5 g/dl	3.5-5.0 g/dl
Aspartate aminotransferase	40 U/l	<50 U/l
Alanine aminotransferase	34 U/l	17-59 U/l
Total bilirubin	0.6 mg/dl	0.2-1.3 mg/dl
Sodium	139 mmol/L	137-145 mmol/L
Potassium	4.4 mmol/L	3.5-5.1 mmol/L
Urea	24 mg/dl	15-36 mg/dl
Random blood sugar	92 mg/dl	70-150 mg/dl
HbA1c	4.4%	<6%
Urine exam (routine)	No pus cells seen	-

**Figure 3:**
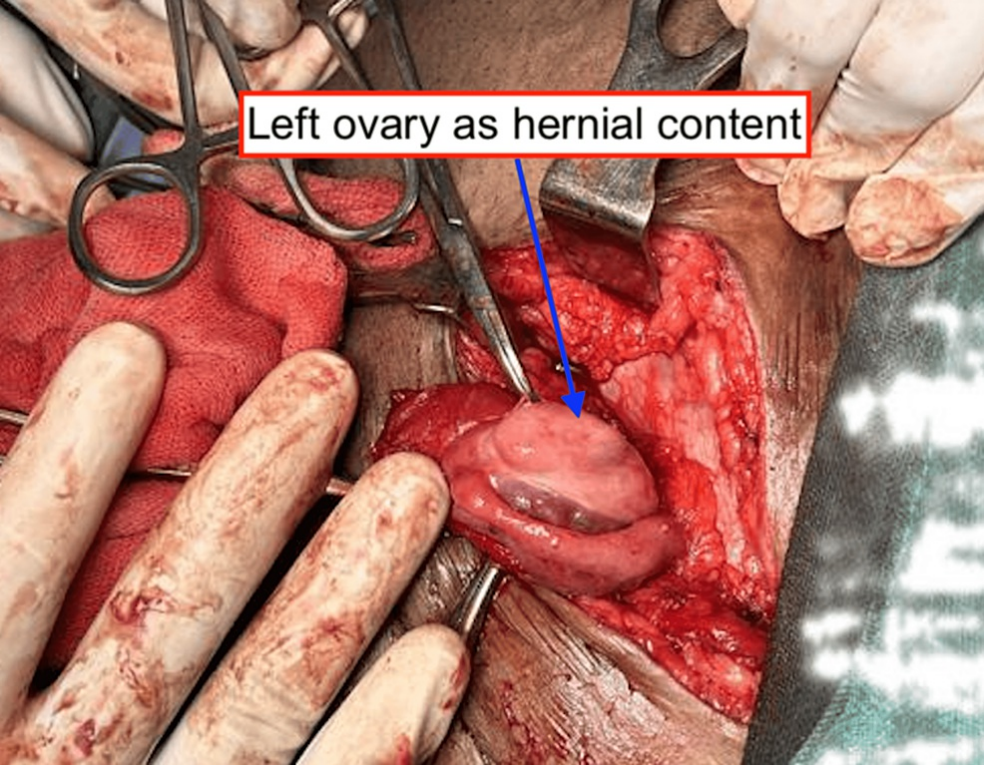
The hernia content, specifically the left ovary, protruding through the defect and covered by the hernial sac

After the anesthetist's fitness had been obtained, the patient was posted for left-sided inguinal hernioplasty. Intraoperatively, the ovary was visualized protruding through the defect covered by the hernial sac (Figure 3).

**Figure 4:**
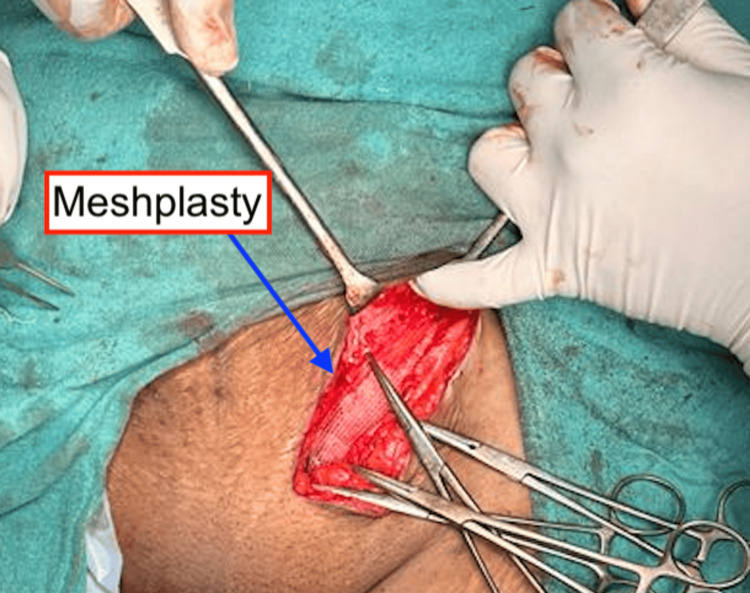
Polypropylene mesh was fixed over the defect as a reinforcement to prevent future recurrence

The sac and the ovary were separated from the surrounding adhesions and reduced back into the abdominal cavity. The defect is repaired using Prolene 2-0 and interrupted sutures. Polypropylene mesh was fixed over the defect as a reinforcement to prevent future recurrence (Figure 4).

The incision was closed in layers, and the procedure was uneventful. Postoperatively, the patient has been administered intravenous fluids, IV antibiotics, and analgesics. Judicious vital monitoring was done. Postoperatively, the patient is clinically and vitally stable with a healthy suture line.

## Discussion

The presented case of a 37-year-old female with an inguinal hernia containing the left ovary with concomitant incidental finding of an ectopic, malpositioned left pelvic kidney unveils a rare confluence of medical anomalies, challenging the conventional expectations associated with inguinal hernias.

Inguinal hernias are a common and usually benign condition in the general population. Repairing inguinal hernias is a significant part of frequently performed surgical procedures [6]. Sometimes, unique structures within the abdominal area may be discovered within a hernial sac. Gynecological elements like ovaries, fallopian tubes, ligaments, and the uterus have been identified as infrequent contents of an inguinal hernial sac. These occurrences are more prevalent in female infants and young girls, making up around 6-7% of inguinal hernias in this specific patient subgroup. Such findings are often linked to irregular development in the genital tract. Although the presence of an indirect hernia, often trapped and unable to be pushed back, containing an ovary might not be rare in young girls, it is less frequently reported in women of reproductive age. In women of reproductive age presenting with a groin mass, it is crucial to consider ovarian inguinal hernia as a potential differential diagnosis. Special attention must be given to the timely and accurate preoperative diagnosis of a groin mass in this specific patient subgroup to prevent delays in diagnosis and avoid complications such as ovarian torsion or damage. Since 88% of patients displayed a lump or swelling in the inguinal area, it's crucial to thoughtfully assess the viability of gynecological structures and the difficulties linked to relocating them inside the peritoneal cavity during the restoration process.

The diagnosis of inguinal hernias is typically established through clinical examination, with the patient's signs and symptoms serving as key indicators. In cases of strangulated and obstructed inguinal hernias, the nature and severity of pain in the mass region can aid in differentiation [7]. Additionally, radiological investigations can complement the diagnostic process for ovarian inguinal hernias. Early utilization of ultrasound evaluation, including color Doppler ultrasound, can provide valuable insights into the nature of the herniated structures and their blood supply. This is particularly beneficial in distinguishing between obstructed and strangulated hernias, such as ovarian torsion [8].

If the diagnosis is unclear or if there are concerns about additional complications. Other differential diagnoses for a groin bulge in females include lymphadenopathy, lymphoma, metastatic neoplasm, abscess, hematoma, femoral artery aneurysm, etc. If there are concerns about ovarian torsion, additional tests such as ovarian Doppler ultrasonography may be considered to assess blood flow to the ovary. It is noteworthy that the diagnostic strategy may differ depending on the distinct features of the case and the resources accessible in the medical setting. A multidisciplinary approach is recommended in challenging scenarios to ensure a comprehensive and accurate diagnosis.

The treatment of inguinal hernias involving gynecological structures, such as the ovary, typically involves a combination of conservative management and surgical intervention. The approach may vary based on the severity of symptoms, the presence of complications, and the patient's overall health. Surgical repair is often recommended for inguinal hernias containing gynecological structures, particularly if they are symptomatic, at risk of incarceration, or associated with complications such as ovarian torsion.

Surgical techniques

*Herniorrhaphy* 

Herniorrhaphy is a surgical technique in which the surgeon utilizes sutures to close the hernia defect, relying on native tissue for the repair. This approach is particularly suitable in cases where the operative field is contaminated or in emergency surgeries where the viability of the hernia contents is uncertain. The meticulous sewing of the weakened tissue aims to reinforce the area, effectively repairing the hernia and restoring the integrity of the abdominal wall. Herniorrhaphy is typically chosen when the hernia is smaller, and the use of mesh is not deemed necessary.

Hernioplasty

Hernioplasty employs a tension-free approach using a mesh to reinforce the weakened area, significantly reducing the risk of recurrence. The use of a synthetic mesh, typically composed of durable materials, is strategically placed over the hernia defect, providing substantial strength to the abdominal wall. Prosthetic repairs, characterized by their tension-free nature, are associated with a lower hernia recurrence rate compared to tissue repairs. This approach enhances the overall durability and effectiveness of the hernia repair, minimizing the likelihood of recurrence and optimizing patient outcomes.

Laparoscopic Repair

Minimally invasive techniques, such as laparoscopic surgery, offer advantages like smaller incisions, reduced postoperative pain, and quicker recovery. Laparoscopic repair is often recommended for select cases, including smaller hernias and instances where a minimally invasive approach is preferred. Two common laparoscopic procedures include the transabdominal preperitoneal procedure and the total extraperitoneal procedure, each offering unique benefits in certain clinical scenarios. These advanced techniques involve accessing the hernia site through laparoscopic means, providing additional options for surgeons to tailor the approach based on the specific characteristics of the hernia and the patient's condition.

Discovering the ovary in the hernia raised concerns about fertility, so during the surgery, we took extra care to put it back in its proper place to protect the possibility of fertility. Fertility considerations in cases where the ovary is involved in an inguinal hernia are crucial, particularly in women of reproductive age. The primary goal during surgery is to preserve the ovary and its function, recognizing its significance in reproductive health by the preservation of ovarian blood supply, repositioning of the ovary, and avoidance of ovarian torsion.

Dealing with an ectopic pelvic kidney that wasn't where it should be added another layer of complexity, requiring careful planning by a team of different specialists.

As clinicians encounter similar cases, the insights presented in this discussion aim to guide future diagnostic and therapeutic approaches. The emphasis on patient-centered care, fertility preservation, and the intricacies of surgical intervention reflects the evolving landscape of healthcare, where individualized and holistic approaches are paramount. Ultimately, this case report serves not only to document a rare clinical presentation but also to contribute to the collective knowledge that informs evidence-based practice.

## Conclusions

In conclusion, this case report presents a unique clinical scenario in a 37-year-old woman, where an inguinal hernia, ovarian involvement, and an ectopic pelvic kidney converged. The diagnostic journey, marked by a combination of clinical examination and advanced imaging modalities, underscores the importance of a comprehensive approach to unravel the nuances of such cases. During surgery, the successful repositioning of the ovary was achieved, with a dedicated focus on fertility preservation, accentuating the significance of evidence-based and patient-centered care. The multi-disciplinary team approach played a crucial role in navigating the complexities of this case. In the broader context, this report contributes to our understanding of managing complex hernia cases, emphasizing the uniqueness of each patient. The limited prevalence of such cases in medical records highlights the necessity for ongoing research and discussions in the medical community. Ultimately, this report serves as a guide for healthcare professionals who may encounter similar situations. By sharing our insights, we aim to contribute to the continuous effort to enhance the management of intricate medical cases, ensuring the delivery of optimal care for every patient.
